# Germanium-embedded fabrics attenuate oxidative stress and modulate cytokine activity in a progressive *in vitro* model of endothelial dysfunction

**DOI:** 10.3389/fbioe.2026.1849861

**Published:** 2026-06-12

**Authors:** Saveria Batti, Erwin Pavel Lamparelli, Mariacristina Arianna, Federica Montella, Gina Myers, Nicola Maffulli, Giovanna Della Porta

**Affiliations:** 1 Translational and NanoMedicine Laboratory, Department of Medicine, Surgery and Dentistry, University of Salerno, Baronissi, Salerno, Italy; 2 Incrediwear Holdings, Inc., Chico, CA, United States; 3 Department of Trauma and Orthopaedics, Faculty of Medicine and Psychology, Sant'Andrea Hospital, Sapienza University, Rome, Italy; 4 Centre for Sports and Exercise Medicine, Barts and the London School of Medicine and Dentistry, Queen Mary University of London, London, United Kingdom; 5 School of Pharmacy and Bioengineering, Keele University, Staffordshire, United Kingdom; 6 Research Centre for Biomaterials BIONAM, University of Salerno, Fisciano, Salerno, Italy

**Keywords:** 3D bioprinting, cytokines, endothelial dysfunction, germanium-embedded fabrics, HUVEC, oxidative stress, reactive oxygen species, THP-1

## Abstract

Germanium-embedded fabrics (GEF) have been proposed as bioactive wearable materials capable to modulate inflammatory and oxidative processes through the thermally induced emission of mid- and far-infrared (MIR/FIR) radiation and the generation of negative air ions (NAIs). However, the events governing the inflammatory crosstalk between endothelial and monocytic cells under GEF exposure, particularly regarding the regulation of adhesion molecules like ICAM-1, as well as overall cytokine secretion and NOx production, has been never investigated. This study systematically investigated the effects of GEF on endothelial and monocytic cells in three sequentially *in vitro* culture models assembled to simulate endothelial dysfunction: (i) conventional 2D monolayer single cultures, (ii) 2D static co-cultures, and (iii) a novel biofabricated 3D dynamic perfusion co-culture system, not previously described in the literature. Human umbilical vein endothelial cells (HUVECs) and monocytic THP-1 cells were stimulated with tumour necrosis factor-alpha (TNF-α; 10 ng/mL) and lipopolysaccharide (LPS; 10 ng/mL), respectively, for 3 days to generate reproducible inflammatory baselines prior to GEF exposure (NAI measured at 1,420 ± 145 ions/cm^3^). In single monolayer cultures, GEF did not compromise cell viability, and significantly reduced intracellular reactive oxygen species (ROS) in HUVECs by 37% at day 7 (*p* < 0.01) and modulated cytokine secretion, decreasing interleukin-6 (IL-6) release by 30% and enhancing IL-2 production in THP-1 cells by 4.3-fold (*p* < 0.05). In 2D static co-cultures, GEF consistently reduced endothelial ROS and exhibited biphasic regulation of intercellular adhesion molecule-1 (ICAM-1) expression, with early upregulation followed by significant attenuation at later time points. Cytokine profiling revealed a transient, temporally balanced modulation of both pro-inflammatory (IL-6, IL-8) and anti-inflammatory (IL-10, IL-4) mediators. In the 3D perfused co-culture system, encompassing HUVECs and THP-1 cells bioprinted within methacrylated collagen (ColMA) hydrogels, GEF preserved high cell viability, limited ROS accumulation, and promoted nitric oxide (NO) production, most prominently at day 7. Cytokine profiling in the 3D model confirmed suppression of pro-inflammatory mediators at concentrations orders of magnitude lower than those observed in equivalent 2D co-cultures. GEF exerts a context-dependent, biphasic modulatory action on endothelial inflammation: attenuating oxidative stress and endothelial activation rather than effecting simple immunosuppression. The convergent antioxidant and cytokine-modulating effects across progressively biomimetic culture models support the potential of GEF as a non-invasive, biophysical strategy for the management of vascular inflammatory conditions.

## 1 Introduction

Inflammation represents a central biological response to tissue injury, infection, and metabolic disturbance, coordinating the restoration of homeostasis through complex cellular and molecular cascades (Medzhitov, 2008; Maskrey et al., 2011). Although acutely protective, an inadequately resolved or chronically sustained inflammatory response drives endothelial dysfunction, oxidative stress, and tissue degeneration—key pathophysiological determinants of a spectrum of chronic diseases including rheumatoid arthritis, osteoarthritis, tendinopathy, and cardiovascular disorders (Nathan, 2002; Pacinella et al., 2022; Naderi-Meshkin and Setyaningsih, 2024). Endothelial dysfunction occupies a pivotal position in this continuum, characterised by increased vascular permeability, leukocyte adhesion, and the overexpression of pro-inflammatory mediators, including tumour necrosis factor-alpha (TNF-α), interleukin-1β (IL-1β), and reactive oxygen species (ROS) (Pober and Sessa, 2015). Pharmacological management of chronic inflammation has traditionally relied on corticosteroids and non-steroidal anti-inflammatory drugs (NSAIDs); however, the long-term use of these agents is constrained by systemic adverse effects, tolerance, and limited disease-modifying capacity. These limitations have promoted growing interest in non-pharmacological, non-invasive therapeutic strategies (Meena et al., 2023; Yang et al., 2025).

Among the emerging candidates, functional and bioactive textiles have attracted considerable attention for their potential to modulate inflammation, oxidative stress, and tissue repair through physical rather than chemical mechanisms ((Meena et al., 2023). These materials incorporate semiconductor elements, including germanium, tourmaline, titanium dioxide, and carbonised charcoal, within polymeric fibre matrices, and are reported to emit negative ions and infrared radiation upon activation by ambient or body temperatures above 32 °C (Chen et al., 2022). The resulting production of negative air ions (NAIs) and electromagnetic fields (EMFs) has been associated with measurable biological and clinical effects, as enhanced microcirculation, attenuation of oxidative cascades, and suppression of pro-inflammatory signalling (Marino et al., 2019; Jedrzejczak-Silicka et al., 2021; Jan et al., 2023; Duranti et al., 2024).

Germanium-embedded fabrics (GEF) represent one of the most extensively investigated platforms within this class. Germanium is a Group IV metalloid semiconductor whose electrical resistance decreases with rising temperature, facilitating thermally driven charge-carrier mobilisation (Juho et al., 2021). Upon thermal activation at skin temperature (∼32 °C), germanium-embedded fibres initiate two principal physical processes: (i) the generation of negatively charged oxygen and hydroxyl ions (O_2_^−^, OH^−^) through the interaction of thermally excited surface electrons with atmospheric molecules, and (ii) the emission of mid- and far-infrared (MIR/FIR) electromagnetic radiation in the 2–22 µm wavelength range (Liu et al., 2015; 2024). The generated NAIs have been reported to modulate cellular redox balance by scavenging ROS, suppressing pro-inflammatory signalling pathways including p38 MAPK and AP-1, stimulating endothelial nitric oxide synthase (eNOS) activity, and enhancing nitric oxide (NO) bioavailability to promote microvascular homeostasis (Lin et al., 2015; Kim et al., 2021; Li et al., 2024). In animal studies, NAI exposure reduces blood pressure, attenuates DNA oxidative damage (assessed by 8-OHdG), and downregulates IL-8 and phosphorylated p38 MAPK, collectively substantiating the vascular-protective potential of this modality (Linares et al., 2025).

Concurrently, far-infrared radiation emitted by GEF enhance tissue oxygenation, activate heat-shock proteins, and stimulate cellular metabolism through resonant absorption by water molecules and intracellular biomolecules (MacLachlan et al., 1998). FIR exposure attenuates the expression of endothelial adhesion molecules (VCAM-1, ICAM-1, E-selectin), upregulates cytoprotective enzymes such as haem oxygenase-1 (HO-1), and improves vascular access patency in clinical settings (Vatansever and Hamblin, 2012; Leung, 2015). Additionally, low-intensity magnetic fields (<100 µT) associated with GEF augment enzymatic antioxidant defences, including superoxide dismutase (SOD) and glutathione peroxidase (GPx), thereby enhancing cellular resistance to oxidative insults (Nezamtaheri et al., 2022). These converging physical, chemical, and biological mechanisms collectively position GEF as a multifactorial, non-invasive therapeutic platform capable of addressing key drivers of endothelial dysfunction.

Despite emerging clinical evidence supporting the utility of GEF-based garments in conditions including osteoarthritis, post-surgical recovery, and sports injuries (Marino et al., 2019), the precise cellular and molecular mechanisms underlying their anti-inflammatory activity remain incompletely characterised. Available *in vitro* evidence addressing the direct biological impact of GEF on endothelial cells is particularly sparse. In particular, the role of endothelial–monocytic crosstalk and its regulation through adhesion molecules, cytokine release, and redox signalling has not been systematically investigated. In this study, we address this gap by combining advanced endothelial and monocytic co-culture *in vitro* models under controlled GEF exposure to dissect ICAM-1 regulation, cytokine profiling, and NOx dynamics, thereby providing new mechanistic insight into how GEF modulates inflammatory responses at the vascular interface. Human umbilical vein endothelial cells (HUVECs) and monocytic THP-1 cells were subjected to chemical inflammatory stimulation with TNF-α and LPS, respectively, and co-cultured under three platforms of increasing complexity: conventional 2D monolayer cultures, 2D static co-cultures recapitulating paracrine endothelial–immune interactions, and 3D perfused co-cultures incorporating bioprinted methacrylated collagen (ColMA) hydrogels. Key readouts included cell viability, intracellular ROS generation, ICAM-1 expression, NO production, and multiplex cytokine profiling. This layered approach enables rigorous assessment of GEF bioactivity across model systems of differential physiological fidelity and offers a translational framework for mechanistic interpretation of the clinical effects attributed to this class of wearable materials.

## 2 Materials and methods

### 2.1 Characterisation of germanium-embedded fabric: Surface morphology and ion emission

The surface morphology and microstructure of GEF samples (Incrediwear, Chico, CA, USA), including those subjected to autoclave sterilisation and a single 1-h UV irradiation cycle, were characterised by field-emission scanning electron microscopy (FE-SEM; LEO 1525, Carl Zeiss SMT AG, Oberkochen, Germany). Fabric specimens were sectioned into small pieces, mounted on double-sided carbon adhesive tape affixed to aluminium stubs, vacuum-dried, and coated with a thin chromium layer (∼150 Å) using a turbo sputter coater (K575X, EmiTech, Ashford, UK). Elemental composition was determined by energy-dispersive X-ray (EDX) spectroscopy (INCA Energy 350, Oxford Instruments, Witney, UK) integrated within the SEM system. For all subsequent culture experiments, GEF was precisely weighed and wrapped uniformly around each culture vessel to ensure consistent exposure geometry across experimental conditions (Huang et al., 2025).

Negative air ion (NAI) concentrations were quantified within enclosed incubators (internal volume: 165,000 cm^3^) in the absence and presence of GEF (fabric density: 4.1 × 10^−4^ g/cm^3^ relative to incubator volume). NAI concentrations were measured using a handheld air ion counter (Precis-Ion™ Air Ion Counter Model AIC3Pro; AlphaLab Inc., USA) An equal number of GEF-wrapped flasks was maintained across all experimental setups to standardise ion exposure relative to cell number under both static and dynamic conditions.

GEF is designed to emit mid- and far-infrared radiation (MIR/FIR) within the wavelength range of [4–20 μm], generated by the germanium components. Infrared emission was assessed under physiologically relevant thermal conditions by equilibrating fabric samples on a temperature-controlled plate maintained at 37 °C. Thermal infrared images were acquired using an infrared camera (T70 Thermal Imaging Camera, ULIRVISION). GEF sample was compared with non-functionalized textile control under identical acquisition settings and environmental conditions. Apparent surface temperature and infrared emission profiles were quantitatively analyzed within predefined regions of interest (https://go.getincrediwear.com/cb-tsl)). Baseline NAI concentration within the empty incubator was 85 ± 12 ions/cm^3^; introduction of GEF elevated this to 1,420 ± 145 ions/cm^3^ (Supplementary Figure S1), confirming substantial ion enrichment attributable to GEF activation.

### 2.2 Cell culture

Primary human umbilical vein endothelial cells (HUVECs; Innoprot, Bizkaia, Spain), isolated from healthy donor umbilical veins at passage 1, were expanded in Endothelial Cell Growth Medium (Innoprot) supplemented with 5% foetal bovine serum (FBS), 1% Endothelial Cell Growth Supplement (ECGS), and 1% penicillin/streptomycin. Cells were seeded onto Nunclon™ Supra T-75 flasks (Thermo Fisher Scientific, Waltham, MA, USA) at 5–6 × 10^3^ cells/cm^2^ without additional matrix coating. Human monocytic THP-1 cells (ATCC, Manassas, VA, USA) were maintained in suspension in RPMI-1640 medium (Aurogene, Rome, Italy) supplemented with 10% heat-inactivated FBS (SIAL, South America), 1% penicillin/streptomycin, and 2 mM L-glutamine (Gibco™, Thermo Fisher Scientific), and subcultured every 2–3 days to maintain a density of 2.5–10 × 10^5^ cells/mL. Co-culture medium comprised a 1:1 mixture of RPMI-1640 and Endothelial Cell Medium (ECM), supplemented with 5% FBS, 2 mM L-glutamine, 1% penicillin/streptomycin, and 0.5% ECGS.

To provide a comparative baseline for the 3D bioprinted model, a 2D co-culture was established in 12-well plates. HUVECs and THP-1 cells were seeded at the same 1:2 ratio used for 3D constructs. These 2D cultures were maintained in the shared co-culture medium and subjected to the same experimental conditions and pro-inflammatory stimulations (LPS) as the 3D scaffolds. This 2D setup served as a reference to normalize and validate the cellular responses across all analyzed parameters.

Three-dimensional constructs were fabricated using a INVIVO 4D2D bioprinter (Rokit Healthcare, Seoul, South Korea). Methacrylated collagen (ColMA; PhotoCol®, Advanced BioMatrix, Carlsbad, CA, USA) at 8% (w/v) was incubated at 37 °C to optimise viscosity prior to incorporation of 0.25% lithium phenyl(2,4,6-trimethylbenzoyl) phosphinate (LAP) photoinitiator. HUVECs and THP-1 cells were co-embedded at a 1:2 ratio within ColMA bioink at a total density of 1.5 × 10^6^ cells/mL. Scaffolds (dimensions: 2 mm height × 5 mm width) were cultured for 7 days within a previously validated perfusion bioreactor (Cortella et al., 2025) — a 12-well plate equipped with silicone tubing connected to a peristaltic pump delivering continuous medium flow at 1 mL/min.

Control conditions were defined as follows: in cytotoxicity assays (Figure 3), CTR + refers to cells treated with 10% ethanol and CTR− to untreated cells. In inflammatory assays (Figures 4, 8), CTR− corresponds to cells stimulated with LPS/TNF-α without GEF treatment. To analyze HUVECs and THP-1 cells independently within the 2D co-culture system, a separation protocol based on differential cell adhesion was employed. At each experimental time point, the culture medium containing the non-adherent THP-1 cells was aspired and centrifuged. The adherent HUVEC monolayer was subsequently washed three times with phosphate-buffered saline (PBS) to remove any residual monocytes before proceeding with cell-specific downstream analyses. In contrast, for the 3D model, cells were analyzed *in situ* within the ColMA hydrogel to evaluate the integrated response of the co-culture environment.

### 2.3 Cell viability assays

For 2D cultures, HUVECs (passage 3; 7–8 × 10^3^ cells/cm^2^) were stimulated with TNF-α (Sigma-Aldrich, Milan, Italy) and THP-1 cells (1.0 × 10^6^ cells/mL) with lipopolysaccharide (LPS; Sigma-Aldrich). At each designated time point, metabolic activity was assessed using the MTT (3-(4,5-dimethylthiazol-2-yl)-2,5-diphenyltetrazolium bromide) assay: cells were incubated with MTT solution (0.5 mg/mL) for 4 h at 37 °C in 5% CO_2_, after which formazan crystals were dissolved in dimethyl sulfoxide (DMSO) and absorbance measured at 570 nm (TECAN Infinite M200 Pro, Männedorf, Switzerland). Cell viability was expressed as a percentage relative to untreated controls according to Equation 1:

$$Dehydrogenase\;activity\left(\%\right)\;=\;\left[\frac{\left(Abstreated\;-\;Absblank\right)}{\left(Absuntreated\;-\;Absblank\right)}\right]\;\times \;100$$ (1)

For 3D constructs, viability was assessed using the Live/Dead Cell Viability Kit (Sigma-Aldrich), combining Calcein AM and propidium iodide (PI) at a 2:1 ratio (Scala et al., 2023). Scaffolds collected at days 0, 3, and seven were washed three times with 1× PBS, incubated with the staining reagents for 15 min at 37 °C, washed again, and imaged at ×4 and ×10 magnification by fluorescence microscopy (Eclipse Ti, Nikon, Tokyo, Japan). Day 0 assessments confirmed immediate post-printing viability (Lamparelli et al., 2021).

### 2.4 Intracellular superoxide detection by dihydroethidium assay

Intracellular superoxide (O_2_^−^•) was quantified using the ROS Detection Cell-Based Assay Kit employing dihydroethidium (DHE; Cayman Chemical, Ann Arbor, MI, USA). In 2D cultures, HUVECs were seeded in black 96-well plates at 6 × 10^3^ cells/well; THP-1 cells at 7.5 × 10^4^ cells/well were centrifuged to remove medium prior to staining. Both cell types were incubated with DHE for 90 min at 37 °C in the dark, and fluorescence was measured at excitation/emission wavelengths of 500/590 nm (TECAN Infinite M200 Pro). For 3D constructs, scaffolds were stained with 5 µM DHE for 2 h, then fixed with 4% paraformaldehyde (PFA) for 2 h and counterstained with DAPI (1:10,000, 15 min). Confocal images were acquired at ×20 magnification (Leica SP5, Leica Microsystems, Wetzlar, Germany) and analysed using ImageJ (NIH, Bethesda, MD, USA) for fluorescence intensity quantification. ROS levels were expressed as the DHE/DAPI fluorescence ratio.

### 2.5 Protein extraction and Western blot analysis

For ICAM-1 quantification, HUVECs seeded in 12-well plates (4 × 10^5^ cells/well) were lysed at each time point in RIPA buffer supplemented with protease and phosphatase inhibitors. In co-culture experiments, HUVECs and THP-1 cells were separated by centrifugation prior to individual lysis. Protein concentrations were determined by BCA colorimetric assay (Novagen, Darmstadt, Germany) using a bovine serum albumin standard curve. Samples (20 µg protein/lane) were resolved by SDS-PAGE on 4%–20% gradient gels (Bio-Rad, Hercules, CA, USA), transferred to PVDF membranes *via* the TransBlot Turbo system (Bio-Rad), and blocked in 5% non-fat dry milk in PBST (PBS +0.1% Tween-20). Membranes were incubated overnight with primary antibodies, followed by HRP-conjugated secondary antibodies. Antibody details are provided in Table 1. Bands were detected by enhanced chemiluminescence (ChemiDoc MP, Bio-Rad) and densitometrically quantified using ImageJ, normalised to GAPDH.

**TABLE 1:** Primary and secondary antibodies used for Western blot analysis.

Antibody	Supplier	Dilution	Blocking solution
Rabbit anti-ICAM-1	Abcam	1:1000	1% non-fat milk/PBST
Mouse anti-GAPDH	ProteinTech	1:1000	5% non-fat milk/PBST
Anti-rabbit IgG–HRP	Sigma-Aldrich	1:3000	5% non-fat milk/PBST
Anti-mouse IgG–HRP	Sigma-Aldrich	1:3000	5% non-fat milk/PBST

ICAM-1: intercellular adhesion molecule-1; GAPDH: glyceraldehyde 3-phosphate dehydrogenase; HRP: horseradish peroxidase; PBST: PBS +0.1% Tween-20.

### 2.6 Nitric oxide quantification

Nitric oxide (NO) production was assessed indirectly by quantifying stable end-products (nitrite and nitrate) in culture supernatants collected at days 3, 5, and 7, using a commercial Nitrate/Nitrite Colorimetric Assay Kit (Cayman Chemical) based on the Griess reaction. Nitrates were first enzymatically reduced to nitrites to permit total NO measurement. Sample dilutions were optimised to ensure values fell within the linear range of the standard curve. Absorbance was measured at 540 nm (TECAN Infinite M200 Pro), and results expressed as µM.

### 2.7 Cytokine quantification

TNF-α release was quantified in culture supernatants by sandwich ELISA (Human TNF-α Confirm ELISA Kit, Millipore, Burlington, MA, USA) per the manufacturer’s instructions. Simultaneous multiplex quantification of ten cytokines (GM-CSF, IFN-γ, IL-1β, IL-2, IL-4, IL-5, IL-6, IL-8, IL-10, and TNF-α) was performed using the Cytokine 10-Plex Human Panel kit (Invitrogen, Thermo Fisher Scientific) on the Luminex™ 200 platform (Luminex Corporation, Austin, TX, USA) (Ciardulli et al., 2020). Concentrations were automatically computed by xPONENT® software (Luminex Corporation) against the assay standard curve.

### 2.8 Statistical analysis

All data are expressed as mean ± standard deviation (SD) from a minimum of three independent experiments, each performed in duplicate or triplicate. Statistical analyses and data visualisation were performed using GraphPad Prism 9 (GraphPad Software, San Diego, CA, USA) and Microsoft Excel. Comparisons between two groups were made using Student’s unpaired t-test; comparisons among multiple groups employed one-way or two-way analysis of variance (ANOVA) followed by post-hoc testing using Sidak’s or Dunnett’s correction, as appropriate. Statistical significance was defined as p < 0.05. Significance thresholds are denoted in figures by asterisks in accordance with GraphPad Prism default conventions.

## 3 Results

### 3.1 Microstructural characterisation of GEF

**FIGURE 1:**
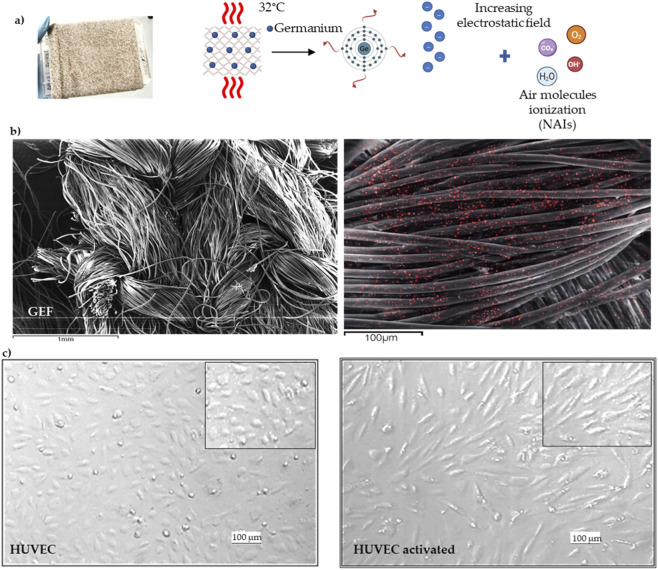
**(a–c)** Description of GEF activity and microscopic characterization of GEF and HUVEC cell. Generic representation of infrared and ion emission at temperatures above 32 °C of GEF textiles **(a)**. Scanning electron microscopy (SEM) images showing the microstructure of the textile fabric, the germanium was embedded in the fibers and was detected (as red dots) by SEM-EDX analysis **(b)**. Optical microscopy (OM) images illustrating non-activated and activated endothelial phenotypes of HUVECs after 3 days of TNF-α stimulation (10 ng/mL) **(c)**.

Scanning electron microscopy revealed a well-defined fibrous microarchitecture in GEF, with individual fibres of uniform diameter (10 μm) and a subtly roughened surface texture consistent with semiconductor particle incorporation. At higher magnification, EDX mapping of the fibre surfaces confirmed the presence of embedded germanium (see red dots), even if was not possible a proper quantification (Figure 1a,b). The interconnected fibre network provides substantial surface area which is expected to facilitate thermally activated ion generation. For contextualisation, optical microscopy of TNF-α-stimulated HUVECs (10 ng/mL, 3 days) demonstrated characteristic morphological changes associated with endothelial activation, including the acquisition of an elongated, spindle-like phenotype, disruption of the cobblestone monolayer architecture, and increased directional alignment, features consistent with inflammatory remodelling (Figure 1c).

### 3.2 Optimisation of pro-inflammatory stimulation conditions

**FIGURE 2:**
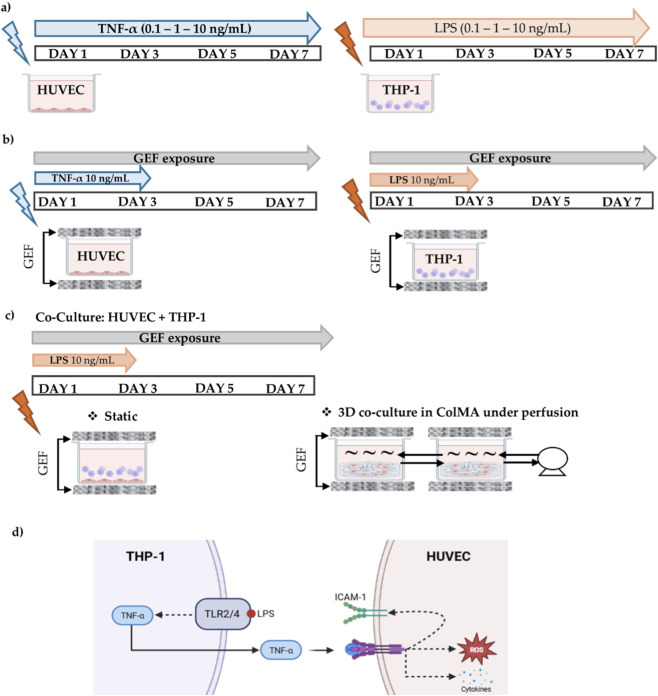
**(a–d)** Experimental design and setup of inflammatory stimulation models using HUVECs and THP-1 cells. Optimization of inflammatory stress conditions applied to HUVECs (TNF-α: 0.1, 1, and 10 ng/mL) and THP-1 cells (LPS: 0.1, 1, and 10 ng/mL) for up to 7 days under static monolayer culture **(a)**. Schematic representation of the timing of stressor application (TNF-α for HUVECs; LPS for THP-1 cells) during static culture with GEF, together with viability, ROS, and ICAM-1 assessments **(b)**. Schematic representation of the co-culture system, showing endothelial activation of HUVECs in the presence of LPS-stimulated THP-1 cells and the timeline of LPS exposure during culture with GEF **(c)**. Schematic illuistration of paracrine stimulation expected to be mediated by LPS-activated THP-1 cells and its effects on HUVECs within the co-culture environment **(d)**.

**FIGURE 3:**
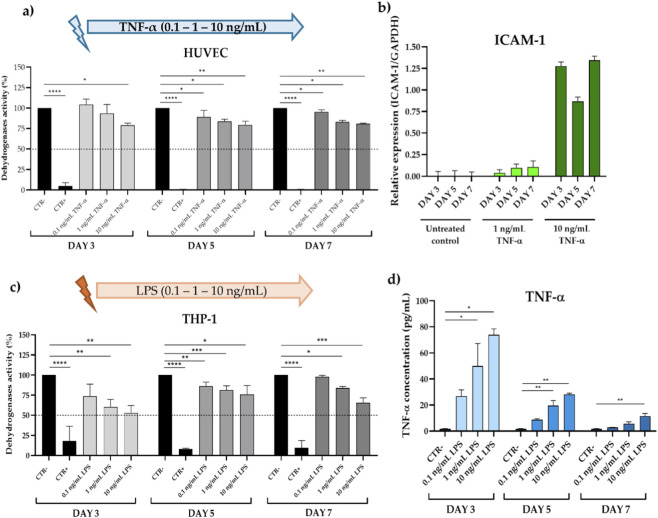
**(a–d)** Effects of TNF-α and LPS stimulation on HUVEC and THP-1 cell responses. HUVEC vi-ability assessed by MTT assay following exposure to LPS at concentrations of 0.1, 1, and 10 ng/mL over 7 days of culture **(a)**, and Western blot analysis of ICAM-1 expression after TNF-α treatment at concentrations of 1 and 10 ng/mL **(b)**. THP-1 cell viability assessed by MTT assay following LPS stimulation **(c)**, and TNF-α expression/secretion induced by LPS, showing a time- and concentration-dependent increase **(d)**. Asterisks indicate statistically significant differences compared with asterisks indicate statistically significant differences compared with negative controls (CTR-; untreated cells without LPS or TNF-α stimulation); 10% ethanol was used as a positive control (CTR+) for cytotoxicity. Data are expressed as mean ± SD. TNF-α secretion by THP-1 cells was quantified by ELISA after LPS stimulation. N = 3; *p ≤ 0.05; **p ≤ 0.01; ***p ≤ 0.001; ****p ≤ 0.0001. Western blot gel data shown in Supplementary Figure S3.

Dose- and time-dependent effects of TNF-α (0.1, 1, and 10 ng/mL) on HUVECs and LPS (0.1, 1, and 10 ng/mL) on THP-1 cells were systematically characterised over 7 days to establish reproducible inflammatory baselines (Figure 2a). In HUVECs, TNF-α treatment produced a concentration-dependent reduction in cell viability, most pronounced at 10 ng/mL by day 3 and at 1 ng/mL by day 7 (Figure 3a). Endothelial activation was confirmed by Western blot analysis of ICAM-1, which displayed a clear concentration-dependent increase as early as day 3, with maximal expression at 10 ng/mL that was sustained through days 5 and 7 (Figure 3b see also Supplementary Figure S3A).

In THP-1 cells, LPS induced a marked, concentration-dependent reduction in viability at day 3, followed by partial recovery at later time points (Figure 3c). TNF-α secretion, quantified by ELISA as a measure of monocytic activation, was significantly elevated at day 3 compared with unstimulated controls, exhibiting the strongest induction at 10 ng/mL. Secretion subsequently declined at days 5 and 7 but remained above baseline levels across all concentrations tested (Figure 3d). Based on these observations, 10 ng/mL TNF-α and 10 ng/mL LPS applied for 3 days were selected as the optimal stimulation conditions for all subsequent GEF experiments, providing robust, non-lethal inflammatory activation consistent with endothelial dysfunction induction.

GEF was wrapped around culture vessels from day 0 to day 7 across all experimental formats (Figures 2b,c). Ion emission measurements confirmed a 17-fold increase in NAI concentration within GEF-treated incubators relative to baseline (1,420 ± 145 vs. 85 ± 12 ions/cm^3^; Supplementary Figure S1). In co-culture experiments, paracrine stimulation of HUVECs by LPS-activated THP-1 cells was considered sufficient to replicate endothelial activation, obviating the requirement for exogenous TNF-α in the co-culture model (Figure 2d).

### 3.3 GEF effects in 2D single monolayer cultures

**FIGURE 4:**
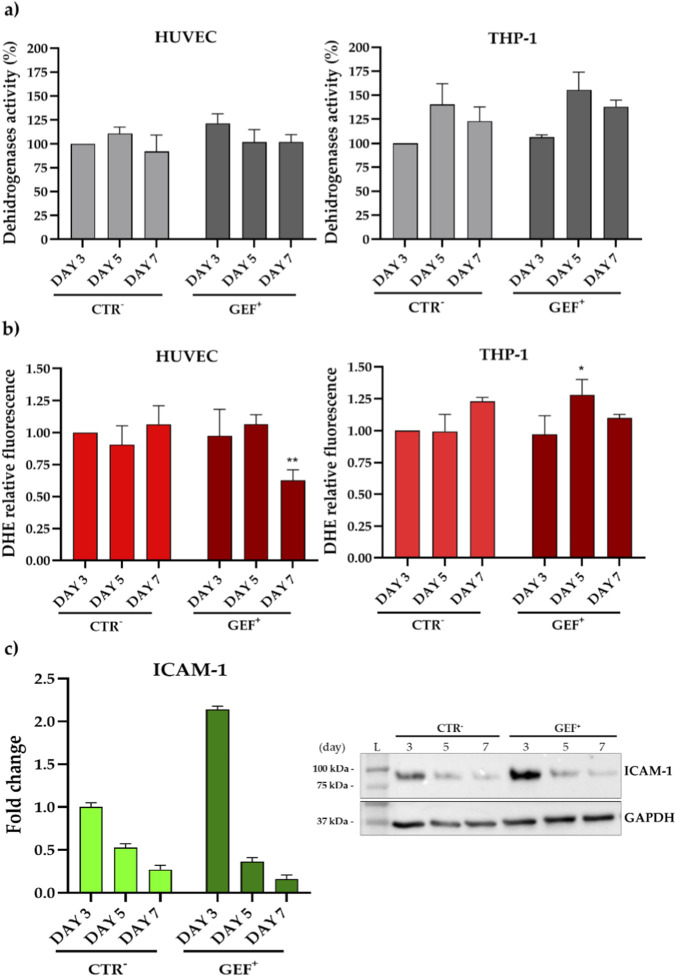
**(a–c)** 2D static single cultures: assessment of GEF activity assayed by cell viability, oxidative stress, and endothelial activation. HUVEC and THP-1 viability was assessed by MTT assay **(a)**. ROS levels measured along the culture; data were normalized to the respective control conditions at day 3 (end of stress application) and are presented as mean ± SD **(b)**. Western blot analysis of ICAM-1 expression in HUVECs after TNF-α stimulation (10 ng/mL for 3 days) showed larger expression reduction in cultures with GEF **(c)**. Negative controls (CTR-) consist of cells stimulated with LPS or TNF-α without GEF treatment. N = 3; *p < 0.05; **p < 0.01.

The effects of GEF were first assessed in individual HUVEC and THP-1 monolayers stimulated with TNF-α and LPS (10 ng/mL), respectively, over a 3-day induction period, followed by evaluation at days 3, 5, and 7. GEF exposure did not significantly affect cell viability in either cell type at any time point, although a trend towards higher viability was noted in THP-1 cells at days 5 and 7 (Figure 4a). In contrast, intracellular ROS levels were significantly reduced in GEF-treated HUVECs at day 7 compared with control cultures (37% reduction; p < 0.01), whereas a statistically significant ROS increase at day 7 was observed in THP-1 cells (Figure 4b). Analysis of ICAM-1 protein expression revealed a biphasic pattern: GEF-treated HUVECs exhibited higher ICAM-1 levels than controls at day 3, followed by a marked and progressive reduction at days 5 and 7, indicating accelerated attenuation of endothelial activation (Figure 4c).

Nitric oxide (NO) and cytokine levels were also evaluated. NO production remained largely unchanged across most conditions, with a significant increase observed only in THP-1 cells at day 3 (Supplementary Figure S3B). Regarding the cytokine profile, a reduction in IL-6 levels was noted in HUVEC at day 7, while an increase in IL-2 was detected in THP-1 cells (Supplementary Figure S4).

### 3.4 GEF effects in 2D static co-culture

**FIGURE 5:**
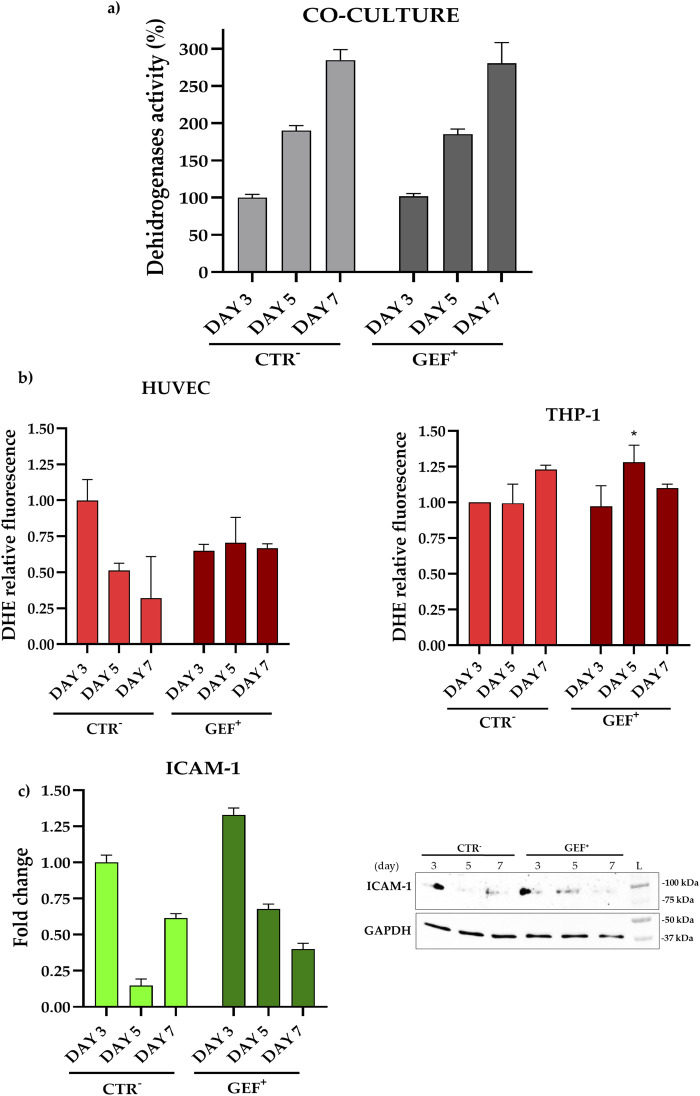
**(a–c)** Stressed 2D static co-cultures: assessment of GEF activity assayed by cell viability, oxidative stress, and endothelial activation. HUVEC: THP-1 co-cultures were established at a 1:2 ratio. Cell viability was assessed by MTT assay following co-culture and LPS stimulation at 10 ng/mL of THP-1 cells, to induce TNF-a secretion **(a)**. Relative ROS levels measured for each cell phenotype in co-culture; data were normalized to the respective control conditions at day 3 (end of stress application) and are presented as mean ± SD **(b)**. ICAM-1 expressed from HUVECs along co-culture **(c)**. Negative controls (CTR-) consist of co-cultures stimulated with LPS without GEF treatment. N = 3; *p < 0.05.

In the HUVEC:THP-1 co-culture system (1:2 seeding ratio), endothelial activation was mediated by paracrine signalling from LPS-stimulated monocytes, as described above. Cell viability in the co-culture was not significantly altered by GEF relative to control conditions (Figure 5a). ROS analysis demonstrated a consistent reduction in intracellular superoxide levels in GEF-treated HUVECs across all time points, reinforcing the antioxidant activity observed in single-culture experiments. In THP-1 cells, GEF treatment was associated with a transient but significant increase in ROS at day 5 (p < 0.05), followed by a return towards baseline by day 7, a pattern not observed in control co-cultures (Figure 5b). Western blot analysis of ICAM-1 in co-cultured HUVECs revealed divergent temporal dynamics: control cultures showed a pronounced increase at day 3, whereas GEF-treated samples displayed elevated ICAM-1 levels at days 3 and 5 relative to controls, followed by a statistically significant reduction at day 7, consistent with GEF-mediated inflammatory modulation (Figure 5c).

### 3.5 GEF effects in 3D dynamic perfusion co-culture

**FIGURE 6:**
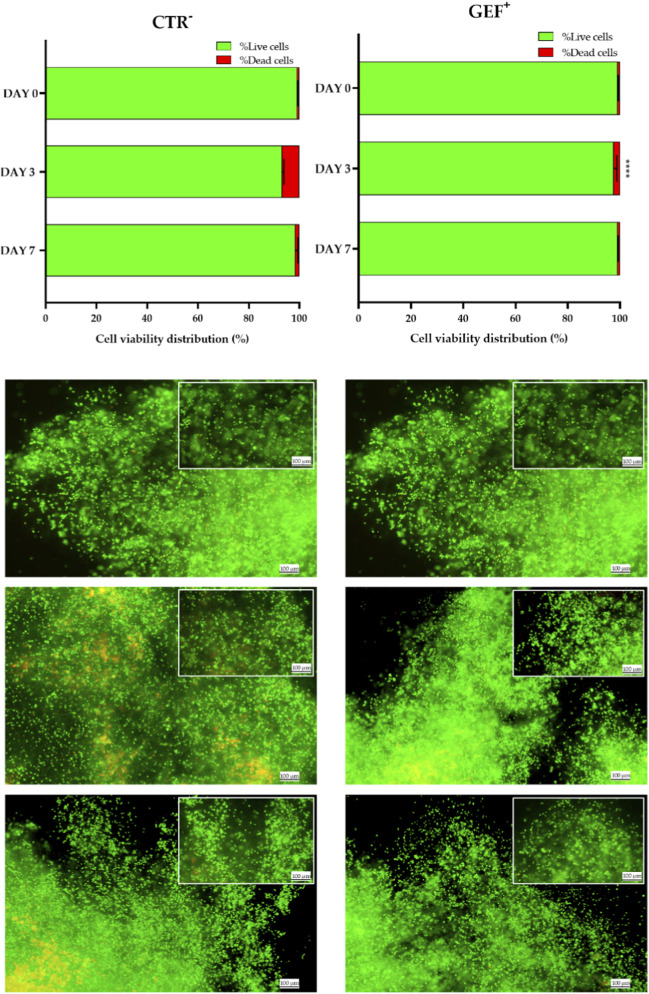
Cell viability within the 3D InRED bioplotted model of co-culture under perfusion with GEF. Cell viability was assessed by Live/Dead assay at days 0, 3, and 7. Data are expressed as mean ± SD; statistical significance is indicated compared with negative controls (CTR-; LPS-stimulated co-cultures without GEF treatment). Data are expressed as mean ± SD; statistical significance is indicated. N = 3 *p < 0.05.

To investigate GEF activity within a physiologically relevant platform, HUVECs and THP-1 cells were co-embedded at a 1:2 ratio within ColMA hydrogel scaffolds (1.5 × 10^6^ cells/mL total) and cultured under continuous perfusion (1 mL/min) with supplementation of LPS for the first 3 days. Live/Dead staining confirmed 99.1% cell viability immediately post-printing, validating the bioprinting protocol (Figure 6, see also Supplementary Figure S5). By day 3, LPS-induced stress reduced viability to 93.3% in control scaffolds, whereas GEF-treated constructs maintained significantly higher viability at 97.6% (p < 0.001). Both groups recovered to comparable high viability by day 7 (98.4% and 99.2% for control and GEF, respectively).

**FIGURE 7:**
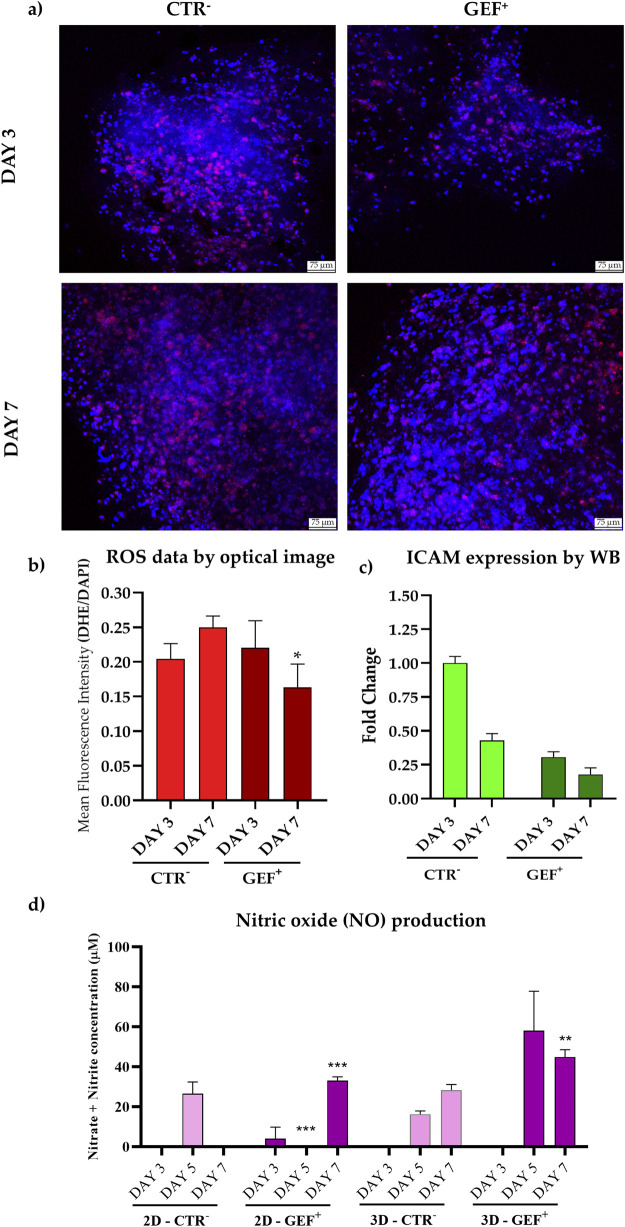
**(a–d)** Evaluation of oxidative stress and cytokine release profile in 3D bioprinted InRED hydrogels under perfusion with GEF. Representative confocal microscope images of 3D culture highlighting the ROS levels quantified as the DHE/DAPI fluorescence ratio in GEF-treated and control culture over time **(a)**. DHE/DAPI ratio plot quantified by image analsisys **(b)**. Western blot ICAM-1 protein quantification in HUVECs along co-culture with and without GEF exposure **(c)**. Nitric oxide (NO) production in 2D and 3D environment expressed as nitrite and nitrate concentration (µM) **(d)**. Negative controls (CTR-) consist of co-cultures stimulated with LPS without GEF treatment. N = 3; *p ≤ 0.05; **p ≤ 0.01; ***p ≤ 0.001.

Oxidative stress in 3D constructs, expressed as the DHE/DAPI fluorescence ratio, was significantly lower in GEF-treated hydrogels at day 7 compared with controls (Figure 7a,b, see also Supplementary Figure S6), consistent with the antioxidant phenotype observed across all model formats. ICAM-1 protein levels, quantified by Western blot, were reduced in GEF-treated 3D culture, with respect to related to controls at both days 3 and 7, with the most pronounced attenuation at day 3 (Figure 7c, see also Supplementary Figure S2B). This observed nice trend, was not properly detectable in 2D culture environment.

NO production, assessed *via* total nitrite/nitrate quantification, revealed model-dependent and GEF-sensitive patterns. In 2D co-cultures, NO was detectable only at day 5 in controls, whereas GEF-treated cultures exhibited measurable levels at days 3, 5, and 7, with consistently higher concentrations at days 3 and 7. In the 3D co-culture, the overall NO secretion was markedly elevated relative to 2D conditions, reflecting the enhanced endothelial functionality of the biomimetic three-dimensional environment. GEF treatment in the 3D model induced a significant further increase in NO production, most pronounced at day 7 (p < 0.01; Figure 7d), suggesting potentiation of eNOS activity under physiologically relevant culture conditions.

### 3.6 Multiplex cytokine profiling

**FIGURE 8:**
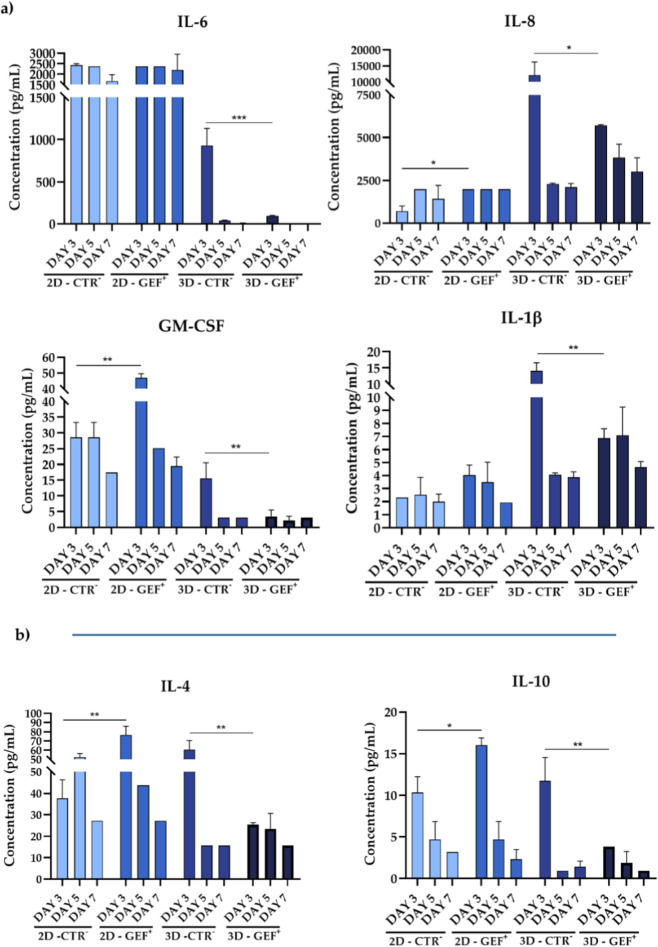
**(a,b)** Cytokine release profiles detected in medium of HUVEC–THP-1 co-cultures in both 2D and 3D environment. Pro-Inflammatory **(a)** and Anti-Inflammatory Cytokine levels **(b)** were quantified in cell culture supernatants following stimulation with and without GEF exposure. Bars represent mean values. Negative control conditions (CTR-) consist of LPS-stimulated cultures without GEF exposure. The histograms are organized into four distinct groups: the first two groups represent the 2D static environment (Control and Treated), followed by the 3D hydrogel cultures, under perfusion (Control and Treated). Pro-inflammatory cytokines (e.g., IL-6, IL-8, GM-CSF, IL-1β) and anti-inflammatory cytokines (e.g., IL-4, IL-10) are shown. N = 3; *p < 0.05; **p < 0.01.

Cytokine concentrations were quantified in culture supernatants from 2D static and 3D perfused co-culture systems, with equivalent cell densities per medium volume across formats. Both pro-inflammatory (IL-6, IL-8, GM-CSF, IL-1β) and anti-inflammatory (IL-4, IL-10) mediators were analysed. In 2D co-cultures, GEF treatment induced cytokine profiles broadly comparable to controls but with a statistically significant increase in anti-inflammatory IL-10 and IL-4 at day 3 (Figures 8a,b), suggesting an early shift towards anti-inflammatory events modulation.

The 3D environment displayed a strikingly distinct cytokine architecture. IL-6 and IL-8 levels in 3D scaffolds were consistently lower compared to both the 2D model and their respective 3D controls, exhibiting a time-dependent decline following an initial peak at day 3. Similarly, GM-CSF and IL-1β levels were significantly lower in GEF-treated 3D cultures than in the corresponding 3D controls at day 3. Notably, IL-4 and IL-10 levels were also substantially lower in GEF-treated 3D cultures at all time points. These findings indicate that the dimensionality and mechanical environment of the culture system profoundly influence the overall pro- and anti-inflammatory cytokine repertoire. Overall, GEF treatment in the 3D co-culture was associated with a suppression of pro-inflammatory cytokines at concentrations orders of magnitude lower than those measured in equivalent 2D co-cultures with GEF, underscoring the superior biomimetic fidelity and GEF-sensitivity of the three-dimensional model.

## 4 Discussion

The present study provides the first systematic, mechanistically grounded *in vitro* characterisation of GEF bioactivity. By moving beyond the limitations of conventional monolayer cultures, this experimental framework enabled a more rigorous and physiologically relevant dissection of GEF-mediated modulation of oxidative stress, endothelial activation, and cytokine dynamics under controlled inflammatory conditions. Previous studies proposed in the literature on GEF activity rely only on conventional monolayer cultures. In this study, we move beyond confirmatory observations by employing a combined endothelial-monocytic model to dissect these mechanisms under controlled conditions. This approach allows us to provide a more integrated and mechanistic view of GEF activity.

A critical preliminary step was the systematic optimisation of inflammatory stimulation conditions. Both TNF-α (HUVECs) and LPS (THP-1 cells) at 10 ng/mL for 3 days elicited robust, reproducible, and non-lethal inflammatory activation, evidenced by ICAM-1 upregulation and monocytic TNF-α secretion, providing a consistent inflammatory baseline from which to interpret GEF effects. The use of activated THP-1 cells as a monocytic compartment is well-established in endothelial co-culture models of vascular inflammation (Meena et al., 2023), and the paracrine crosstalk established in the co-culture system faithfully recapitulates the leukocyte-endothelial interactions operative in chronic inflammatory pathology.

Across all model formats, GEF consistently preserved cell viability and exerted a significant antioxidant effect, most prominently demonstrated by a 37% reduction in intracellular ROS in HUVECs at day 7 in single cultures. This antioxidant activity is mechanistically concordant with the previous described in the literature capacity of NAIs to scavenge free radicals, suppress p38 MAPK and AP-1 activation, and enhance endogenous antioxidant defences including SOD and GPx (Lin et al., 2015; Kim et al., 2021; Li et al., 2024). The reproducibility of the ROS-attenuating effect across 2D and 3D formats, despite the differing oxygen and nutrient transport dynamics of each system, strongly suggests that this represents a genuine and robust biological property of GEF rather than a culture-system artefact.

Of particular mechanistic interest is the biphasic modulation of ICAM-1 expression observed in 2D culture formats. The exposure to GEF was consistently associated with an early upregulation of ICAM-1 at day 3, coincident with the peak inflammatory stimulus, followed by significant attenuation at later time points relative to controls. This temporal pattern suggests that GEF does not simply suppress inflammatory signalling but rather modulates its kinetics, facilitating more rapid transition from the activation to the anti-inflammatory phase. Such a biphasic, modulation-promoting effect is mechanistically distinct from the immunosuppressive profiles of NSAIDs or corticosteroids and is more aligned with the action of anti-inflammatory-phase mediators (resolvins, protectins) that actively programme inflammatory termination (Medzhitov, 2008). GEF activity may be described as a strong inflammation modulation rather than a complete inflammation resolution. In fact, true resolution requires specific cellular hallmarks, such as the induction of macrophage apoptosis or emigration and the cessation of neutrophil infiltration (Fullerton and Gilroy, 2016; Schett and Neurath, 2018), which were not explicitly evaluated in this study. Instead, our findings point toward an accelerated immunomodulatory shift, characterized by a transition from a pro-inflammatory to an anti-inflammatory secretory phenotype. This interpretation is supported by the cytokine profiling data, which revealed transient early increases in anti-inflammatory IL-10 and IL-4 in GEF-treated 2D co-cultures at day 3, while in the 3D model, GEF treatment led to a significant reduction of both pro-inflammatory (IL-6, IL-8, GM-CSF, IL-1β) and anti-inflammatory (IL-4, IL-10) mediators, suggesting a generalized attenuation of the inflammatory secretome in the assembled and more biomimetic environment.

The stimulatory effect of GEF on NO production, particularly in the 3D co-culture model, merits specific discussion. NO is a central mediator of vascular homeostasis: it inhibits platelet aggregation, suppresses leukocyte adhesion molecule expression, and counteracts oxidative stress *via* competitive inhibition of ROS-generating enzymes (Lin et al., 2015). The GEF-dependent enhancement of NO output, most pronounced at day 7 in the 3D system, is consistent with published evidence that both FIR radiation and NAI exposure stimulate eNOS transcription and enzymatic activity (Lin et al., 2015; Leung, 2015). The amplification of this effect in the 3D perfused environment likely reflects the enhanced endothelial phenotypic maturation conferred by three-dimensional scaffold geometry and physiological shear stress (Cortella et al., 2025), conditions known to upregulate eNOS expression. The convergent antioxidant and NO-enhancing effects of GEF therefore suggest a coordinated restoration of vascular homeostasis rather than isolated suppression of individual inflammatory mediators.

A further observation on the proposed 3D environment culture has to be underlined because the 3D co-culture model revealed a notably distinct cytokine landscape relative to its 2D counterpart. This confirms that our novel biofabricated system provides a superior platform for investigating GEF effects, as it captures the restrained cytokine microenvironment characteristic of *in vivo* tissue, which simple monolayer or planar co-cultures fail to recapitulate. Overall pro-inflammatory cytokine levels, particularly IL-6, were substantially lower in 3D constructs, consistent with previously reported observations that three-dimensional biomimetic environments modulate endothelial gene expression programmes and cytokine secretion profiles relative to planar cultures (Cortella et al., 2025). Specifically, GEF exposure significantly reduced IL-6 and IL-8 levels in 3D scaffolds by day 3, a trend not observed in the 2D format where IL-6 remained largely unchanged. The consistent reduction of IL-8 in 3D constructs across conditions may reflect the specific recruitment of CXCR2-dependent signalling pathways that are differentially engaged in three-dimensional *versus* planar geometries. The reduction of IL-10 in the 3D model also suggests that the anti-inflammatory cytokine responses observed in 2D may be culture-system dependent. In the 3D environment, GEF appears to bypass the need for a compensatory anti-inflammatory spike, directly driving the system toward a low-cytokine homeostatic state. Critically, GEF treatment in 3D co-cultures produced pro-inflammatory cytokine concentrations orders of magnitude lower than those measured in analogous 2D co-cultures treated with GEF, further supporting the hypothesis that the 3D system better recapitulates the restrained cytokine microenvironment characteristic of endothelial tissue *in vivo* and thereby reveals the full anti-inflammatory potential of GEF.

The molecular basis of GEF activity likely involves the integrated action of its three principal physical outputs: NAIs, FIR radiation, and low-intensity magnetic fields. At the transcriptional level, these stimuli may collectively suppress NF-κB and AP-1 activation, thereby curtailing TNF-α, IL-6, and IL-8 production; upregulate Nrf2-dependent antioxidant response elements; and enhance eNOS transcription through stabilisation of eNOS mRNA or post-translational phosphorylation of the enzyme (Vatansever and Hamblin, 2012; Lin et al., 2015; Li et al., 2024). The convergent action on multiple molecular targets, rather than on a single pathway, positions GEF as a pleiotropic biophysical modulator and may explain the temporally balanced rather than simply suppressive cytokine profile observed across models. These *in vitro* findings align with, and provide mechanistic context for, previously reported clinical benefits of GEF-based wearable garments in osteoarthritis, post-surgical recovery, and sports injuries (Marino et al., 2019; Justice and Jacob, 2024).

Several limitations should be acknowledged.

To address the mechanistic depth of this work, it must be noted that the present study does not provide direct experimental validation of specific intracellular signaling pathways (e.g., *via* pharmacological inhibition or genetic approaches). Instead, the primary focus was to characterize the overall cellular response to GEF exposure, specifically endothelial-monocytic behavior, ROS modulation, ICAM-1 expression, cytokine secretion, and NOx production, within a controlled experimental framework. The discussion of pathways such as p38 MAPK, Nrf2, and eNOS is therefore intended to provide a biologically plausible context based on existing literature, rather than to imply direct mechanistic validation. Further studies involving targeted pathway analysis will be necessary to define the precise molecular mechanisms underlying the observed effects.

Furthermore, the *in vitro* nature of the models, even in their most complex 3D format, precludes complete recapitulation of the systemic immune milieu, haemodynamic forces, and neurohumoral regulatory inputs operative *in vivo*. The indirect mode of GEF application, wrapping around culture vessels rather than direct skin contact, may underestimate the magnitude of effects achievable under clinical conditions. Additionally, the specific NAI concentrations and FIR flux densities experienced by cells at the microenvironmental level within incubators are difficult to quantify precisely and may differ from those at tissue surfaces *in vivo*. Future investigations should address these constraints by incorporating pathologically relevant primary human cells (e.g., chondrocytes, synovial fibroblasts, tenocytes), applying GEF configurations, elucidating intracellular signalling pathways mediating the observed effects, and, ultimately, validating findings in appropriate animal models.

## 5 Conclusion

This study provides the first mechanistically grounded *in vitro* evidence for the anti-inflammatory and antioxidant bioactivity of germanium-embedded fabrics across three models of increasing physiological complexity. GEF exerted a consistent and reproducible antioxidant effect, attenuating intracellular ROS in HUVECs across all culture formats, and modulated endothelial activation in a biphasic manner, accelerating the transition from ICAM-1 upregulation to anti-inflammatory modulation rather than globally suppressing inflammatory signalling. GEF additionally enhanced NO production in the 3D perfused model, suggesting eNOS stimulation under physiologically relevant conditions.

Despite reported positive clinical *in vivo* outcomes, the mechanisms by which germanium-embedded fabrics (GEF) interact with biochemical pathways reducing inflammation, remain incompletely understood. The observed reduction in intracellular ROS suggests a potential influence on mitochondrial function, possibly *via* modulation of electron transport efficiency. This interpretation is consistent with decreased oxidative stress and the concomitant improvement in nitric oxide (NO) bioavailability. The presence of negative air ions (NAIs) measured in the incubator atmosphere may contribute to the observed effects by modulating extracellular redox balance and attenuating NF-κB–mediated inflammatory signalling. This is supported by the observed reduction in pro-inflammatory cytokines, including IL-6, IL-8, GM-CSF, and IL-1β in the 3D model alongside a precisely tuned modulation of anti-inflammatory mediators, indicating a balanced and adaptive immunomodulatory response rather than a purely suppressive effect. Indeed, cytokine profiling revealed a temporally balanced modulation of pro- and anti-inflammatory mediators, most pronounced in the 3D biomimetic co-culture, which consistently outperformed 2D systems in demonstrating the sensitivity of the inflammatory secretome to GEF-mediated modulation. Collectively, these findings support the candidacy of GEF as a supporting biophysical strategy for the adjunctive management of chronic vascular inflammatory conditions, while delineating a rigorous *in vitro* methodology for future mechanistic and translational investigations in this field.

## Data Availability

The original contributions presented in the study are included in the article/Supplementary Material, further inquiries can be directed to the corresponding author.
