# Supplementary material for: Germanium-embedded fabrics attenuate oxidative stress and modulate cytokine activity in a progressive in vitro model of endothelial dysfunction
Source: Front Bioeng Biotechnol. 2026 Jun 12;14:1849861. doi: 10.3389/fbioe.2026.1849861 (PMC13303586; doi:10.3389/fbioe.2026.1849861)
Supplement: Supplementary file 1 [file DataSheet1.docx]

# Supplementary Figures


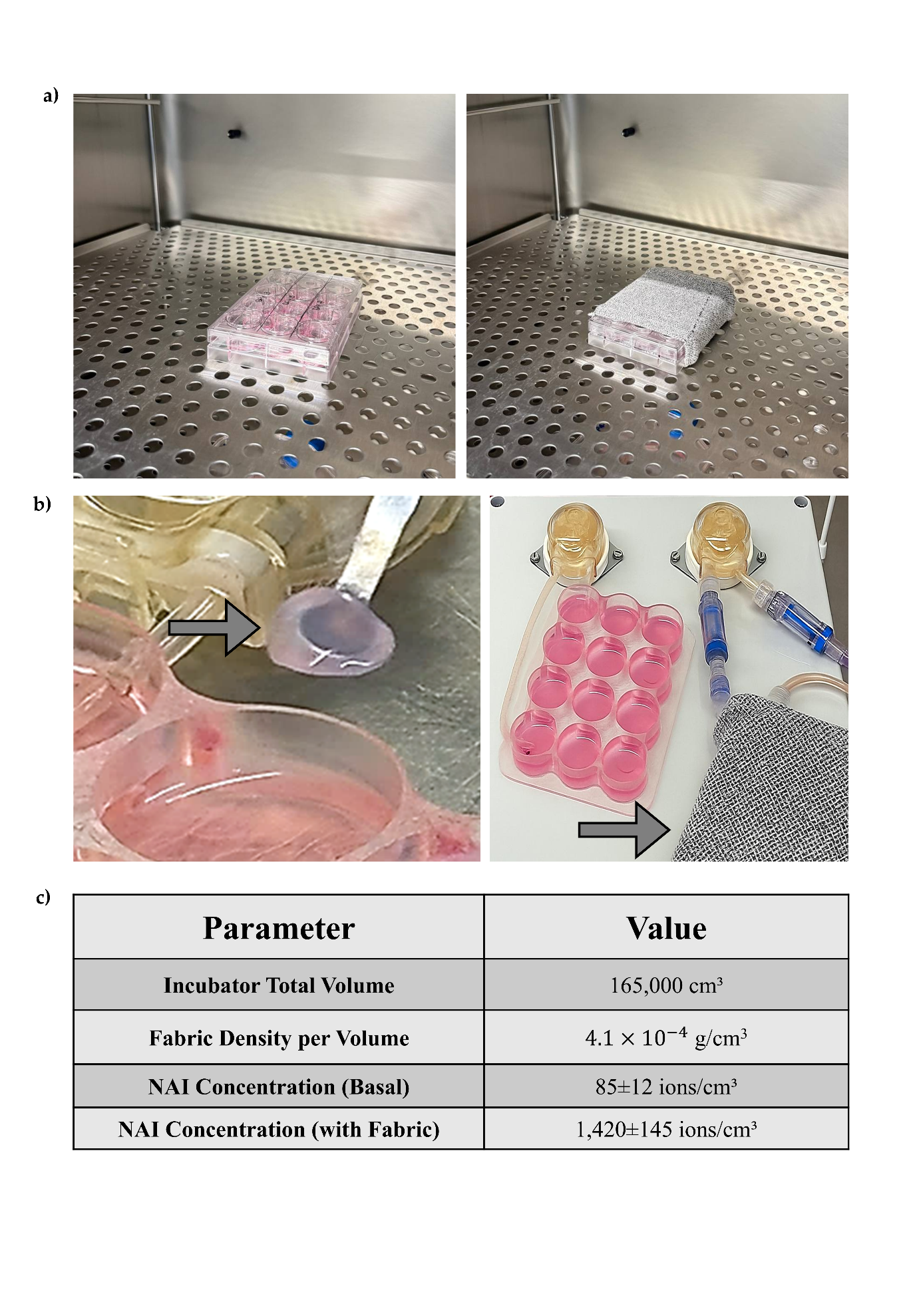


**Figure S1**. Image of 2D vs 3D culture under perfusion within the incubator with and without the GEF textile. Overall Ions were measured by Air Ion Counter (mod. AIC3Pro, www.alphalabinc.com).


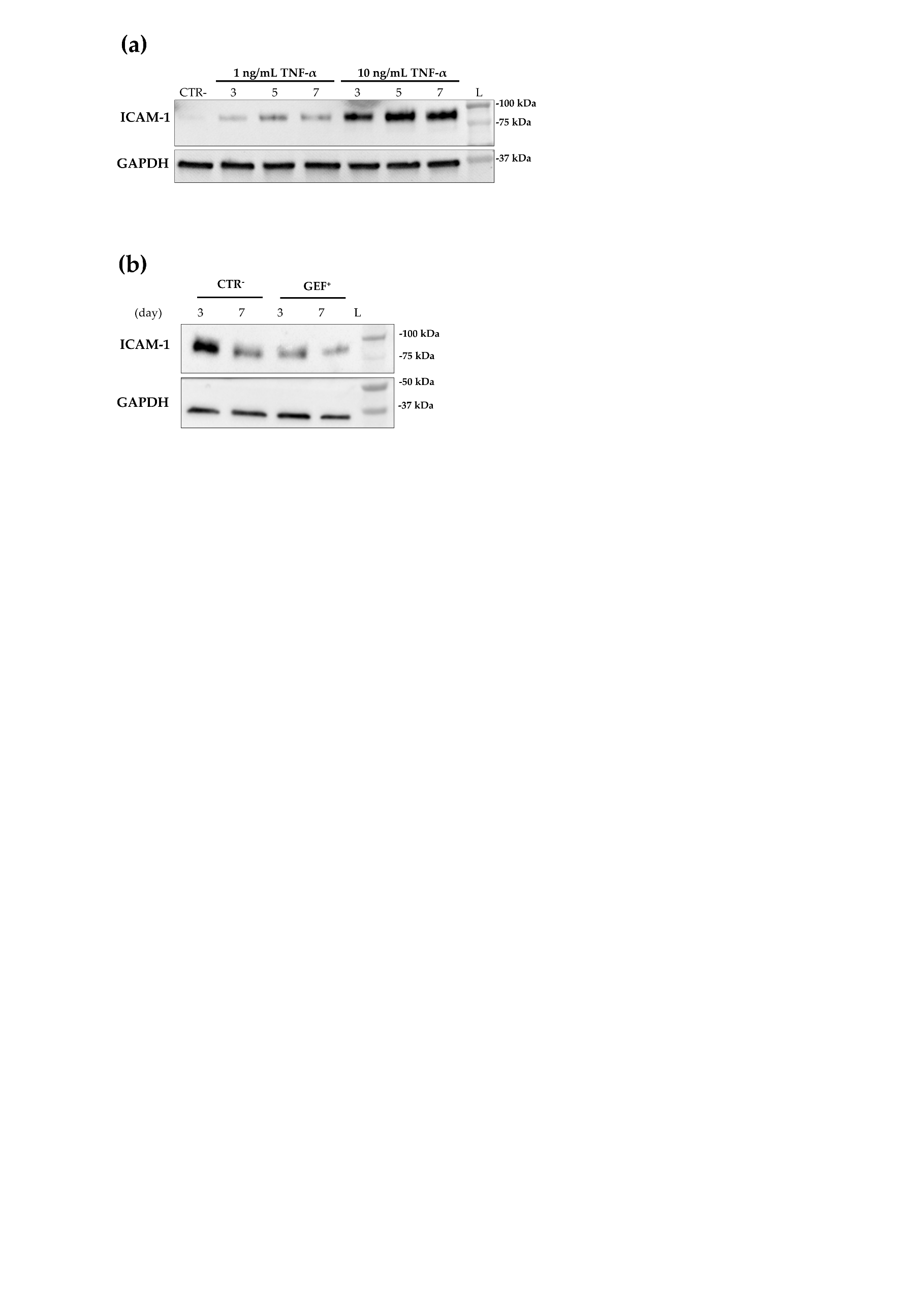


**Figure S2** Full-panel Western Blots for ICAM-1 and GAPDH. Representative immunoblot panels corresponding to the quantitative data presented in Figure 3B (**A**) and Figure 7C (**B**). GAPDH was used as the loading control for protein normalization across all experimental groups.


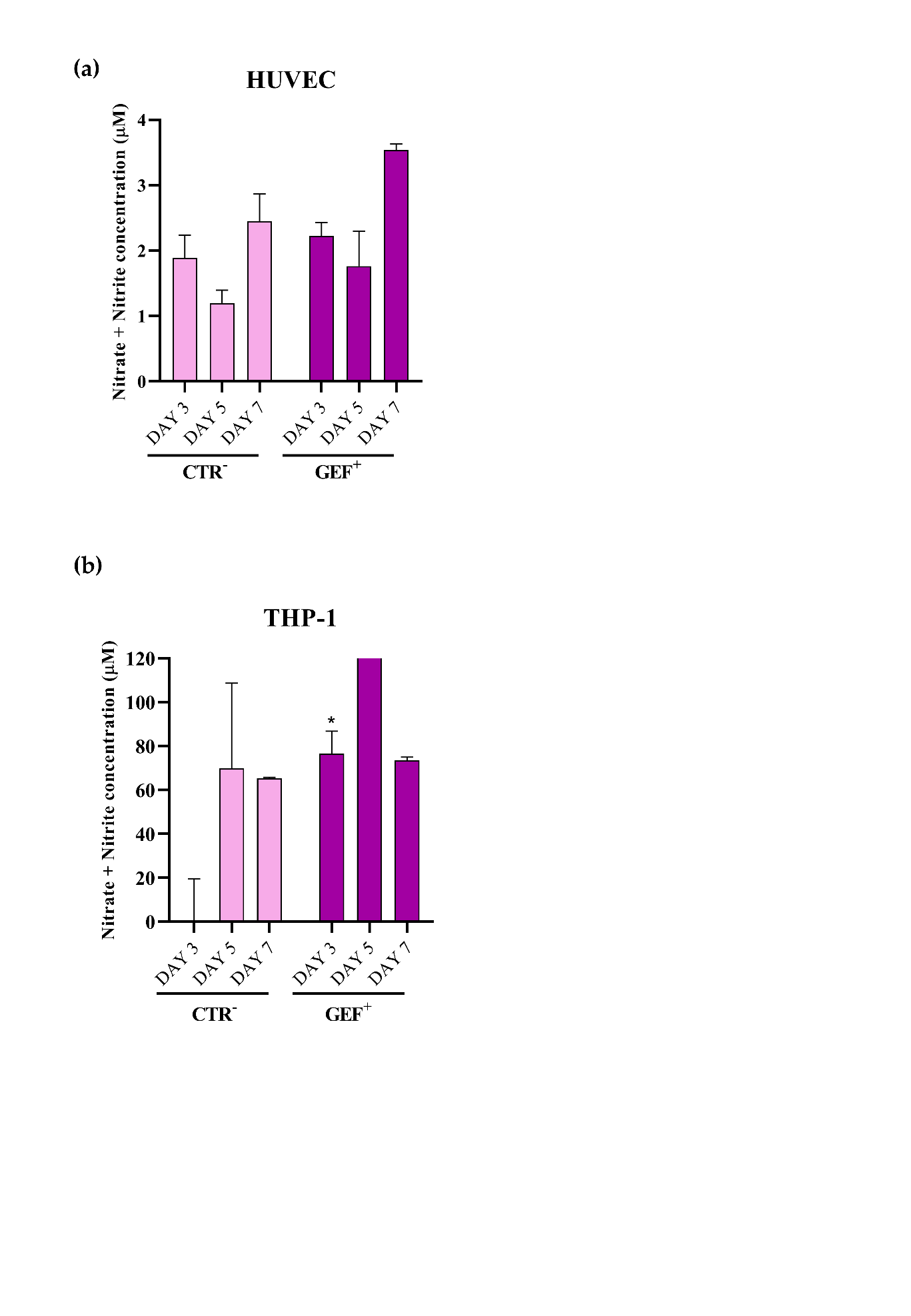


Figure S3. Nitric oxide (NO) production in HUVEC (A) and THP-1 (B) 2D static single cultures expressed as nitrite and nitrate concentration (µM). N=3; ; *p ≤ 0.05.


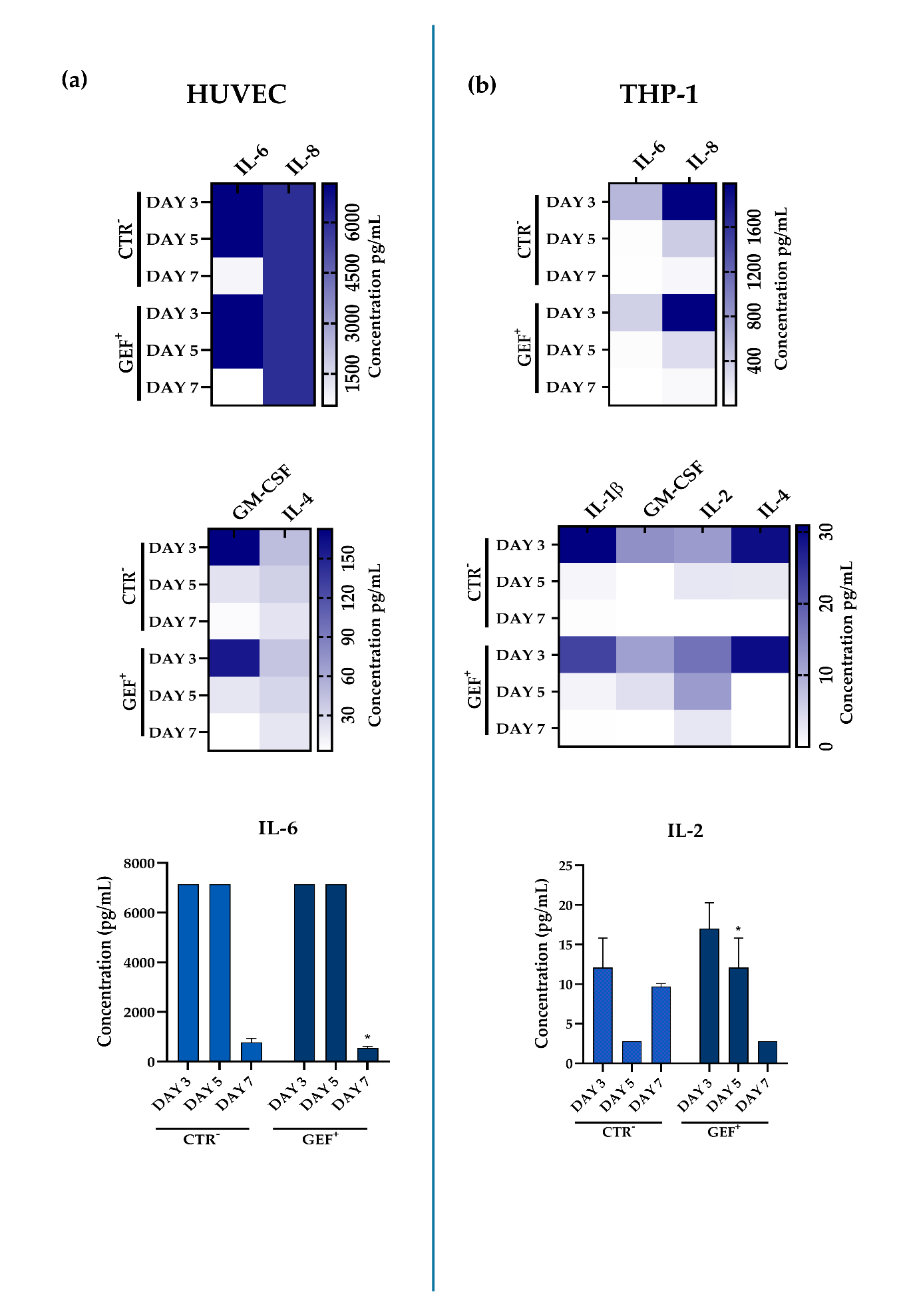


Figure S4. Cytokine release profiles in 2D static monocultures. Cytokine levels were quantified in the supernatants of (a) HUVEC and (b) THP-1 cultures following GEF exposure. Top panels: Representative heatmaps showing the release profiles of IL-6, IL-8, GM-CSF, and IL-4 for HUVEC, and IL-6, IL-8, IL-1β, GM-CSF, IL-2, and IL-4 for THP-1. Bottom panels: Histograms displaying the quantitative variation of IL-6 (HUVEC) and IL-2 (THP-1) levels. N=3; *p < 0.05.

**
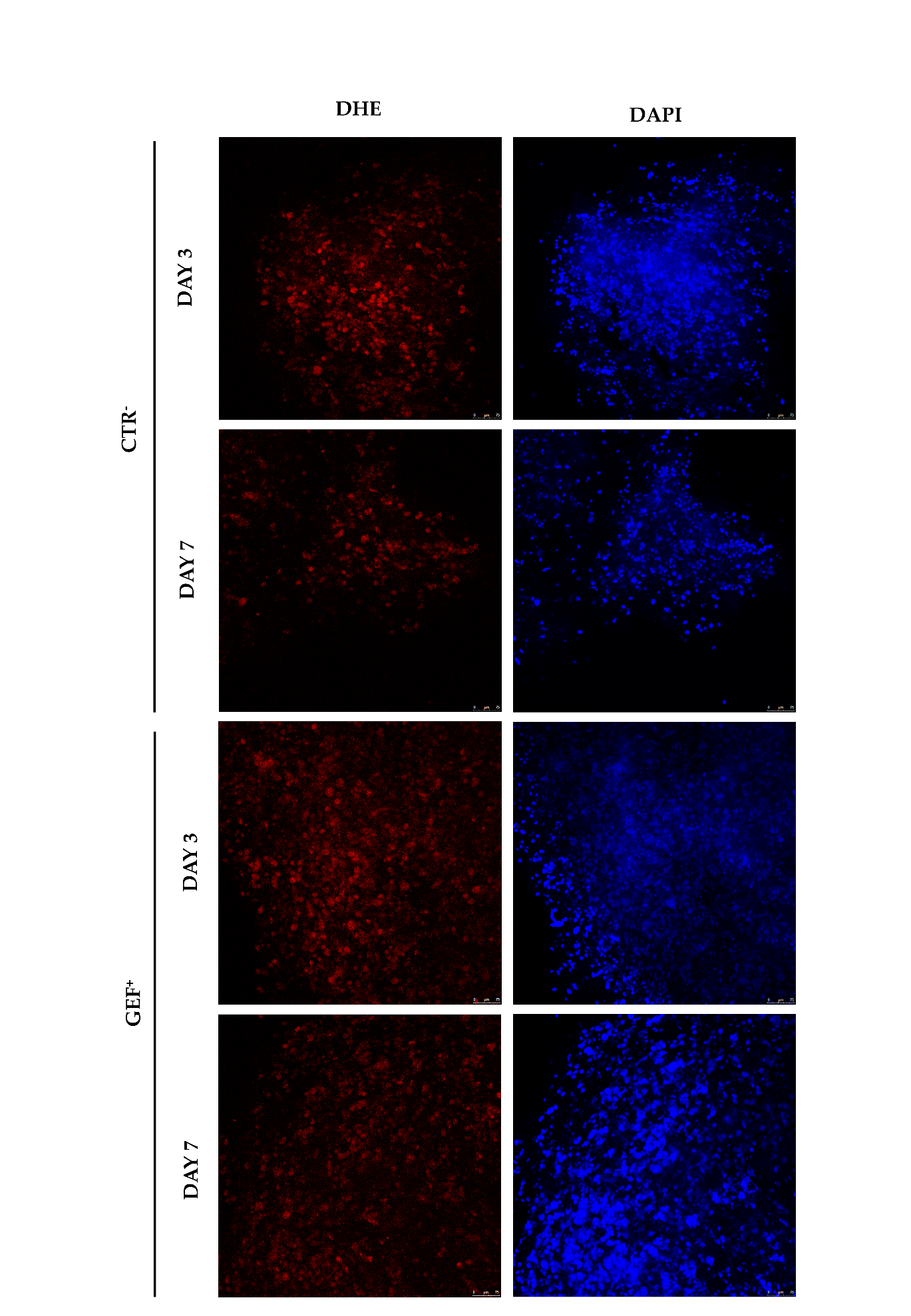
**

Supplementary figure 5. Additional Images of oxidative stress detected in 3D bioprinted scaffold.
